# Functional Characterization of a Novel IRF6 Frameshift Mutation From a Van Der Woude Syndrome Family

**DOI:** 10.3389/fgene.2020.00562

**Published:** 2020-06-04

**Authors:** Mengqi Zhang, Jieni Zhang, Huaxiang Zhao, Vitaly Ievlev, Wenjie Zhong, Wenbin Huang, Robert A. Cornell, Jiuxiang Lin, Feng Chen

**Affiliations:** ^1^Department of Orthodontics, Peking University School and Hospital of Stomatology, Beijing, China; ^2^Department of Anatomy and Cell Biology Carver College of Medicine, University of Iowa, Iowa City, IA, United States; ^3^Central Laboratory, Peking University School and Hospital of Stomatology, Beijing, China

**Keywords:** *IRF6*, Van Der Woude Syndrome, pedigree, *irf6* maternal-null mutant zebrafish embryos, proteasome-dependent degradation

## Abstract

**Background:**

Loss-of-function mutations in interferon regulatory factor-6 (*IRF6*) are responsible for about 70% of cases of Van Der Woude Syndrome (VWS), an autosomal dominant developmental disorder characterized by pits and/or sinuses of the lower lip and cleft lip, cleft palate, or both.

**Methods:**

We collected a Chinese Han VWS pedigree, performed sequencing and screening for the causal gene mutant. Initially, species conservation analysis and homology protein modeling were used to predict the potential pathogenicity of mutations. To test whether a VWS family-derived mutant variant of *IRF6* retained function, we carried out rescue assays in *irf6* maternal-null mutant zebrafish embryos. To assess protein stability, we overexpressed reference and family-variants of IRF6 *in vitro*.

**Results:**

We focused on a VWS family that includes a son with bilateral lip pits, uvula fissa and his father with bilateral cleft lip and palate. After sequencing and screening, a frameshift mutation of *IRF6* was identified as the potential causal variant (NM.006147.3, c.1088-1091delTCTA; p.Ile363ArgfsTer33). The residues in this position are strongly conserved among species and homology modeling suggests the variant alters the protein structure. In *irf6* maternal-null mutant zebrafish embryos the periderm differentiates abnormally and the embryos rupture and die during gastrulation. Injection of mRNA encoding the reference variant of human IRF6, but not of the frame-shift variant, rescued such embryos through gastrulation. Upon overexpression in HEK293FT cells, the IRF6 frame-shift mutant was relatively unstable and was preferentially targeted to the proteasome in comparison to the reference variant.

**Conclusion:**

In this VWS pedigree, a novel frameshift of *IRF6* was identified as the likely causative gene variant. It is a lost function mutation which could not rescue abnormal periderm phenotype in *irf6* maternal-null zebrafish and which causes the protein be unstable through proteasome-dependent degradation.

## Introduction

Van Der Woude Syndrome (VWS, OMIM #119300) is an autosomal dominant developmental disorder characterized by pits and/or sinuses of the lower lip and cleft lip, cleft palate, or both (CL/P, CP) (Van Der Woude, 1954). VWS is the most common form of syndromic orofacial clefting (OFC), accounting for around 2% of all cases, and its phenotype is very similar to the more common non-syndromic forms (Little et al., 2009). Up to now, all causal VWS mutations identified are in either Interferon Regulatory Factor-6 (*IRF6*) (Kondo et al., 2002; Malik et al., 2014; Peyrard-Janvid et al., 2014) or Grainyhead-like 3 (*GRHL3*) (Peyrard-Janvid et al., 2014). Since Kondo et al. (2002) first found a nonsense mutation in *IRF6* in an affected twin and identified mutations in *IRF6* in 45 additional unrelated families affected with VWS, it is now apparent that *IRF6* mutations are responsible for about 70% of cases of VWS (Ghassibe et al., 2004; Malik et al., 2010). Mutations in *GRHL3* account for another 5% of cases of VWS, which may be a new subtype of VWS (VWS2, MIM #606713) (Koillinen et al., 2001; Peyrard-Janvid et al., 2014). Interestingly, in remaining approximately 25% of VWS cases, the causal mutation and causal gene are unknown (Leslie et al., 2016a).

IRF6 belongs to a family of transcription factors. The families share a conserved helix-turn-helix DNA-binding domain (DBD) and a less conserved protein-binding domain named SMIR (Kondo et al., 2002). Popliteal pterygium syndrome (PPS; MIM #119500), also results from mutations in *IRF6* and has a similar orofacial phenotype to VWS but exhibits additional anomalies that include popliteal webbing, pterygia, oral synychiae, adhesions between the eyelids, syndactyly and genital anomalies (Bixler et al., 1973). Mutations that add a termination codon into *IRF6* were reported to be found more often in families with VWS than with PPS (Little et al., 2009). While coding mutations of *IRF6* can cause syndromic OFC, polymorphisms near *IRF6* are overtransmitted in patients with non-syndromic orofacial clefting (NSOFC) (Zucchero et al., 2004). Additional evidence, including linkage analyses, candidate gene analyses, and genome-wide association studies have confirmed the relationship between *IRF6* and NSOFC (Park et al., 2007; Rahimov et al., 2008; Beaty et al., 2010; Sun et al., 2015). We have also reported a rare variant in *IRF6* as likely to be a causative mutation of NSOFC in a pedigree (Zhao et al., 2018a).

IRF6 plays a key role in the formation of periderm, a simple squamous epithelia that comprises the superficial layer of embryonic skin and oral epithelium (Hammond et al., 2019). *Irf6* homozygous null mutant mouse embryos exhibit a defect in epidermal permeability barrier, abnormal skin stratification, and cleft palate with fusion of the palate shelves with the tongue, suggesting a crucial role for the oral epithelium in directing palate development (Ingraham et al., 2006; Richardson et al., 2006). *irf6* maternal-null mutant zebrafish embryos, or wild-type zebrafish embryos injected with mRNA encoding a dominant-negative variant of Irf6 (i.e., lacking the ability to promote transcription), exhibit abnormal periderm differentiation and rupture during gastrulation (Sabel et al., 2009; de la Garza et al., 2013; Li et al., 2017). Interestingly, in the mutant embryos this phenotype is completely rescued upon injection of human *IRF6* mRNA, providing a method to assess the protein function of patient-derived human *IRF6* gene variants (Li et al., 2017).

In this study, we collected a Chinese Han VWS pedigree, performed whole exome sequencing (WES) and screened for the causal gene mutant. We tested the function of this variant by injecting it into maternal-null mutant *irf6* zebrafish embryos. We also examined protein stability and degradation mechanism *in vitro*.

## Materials and Methods

### Participants and DNA Extraction

We recruited a family with inherited VWS at the Peking University Hospital of Stomatology. Informed consent was obtained from each member or his guardian. Peripheral blood samples and clinical information were collected from each family member. The study and the associated research protocols were approved by the ethics committees of the Peking University Hospital of Stomatology (PKUSSIRB-201520012).

A dentist and a maxillofacial surgeon recorded the clinical phenotypes and performed diagnoses. Patients also underwent a complete physical examination to evaluate other organ malformations. Prenatal exposure history of the mother of the proband, including smoking, drinking, medicine and supplement use, disease history, and radiation exposure was recorded.

QIAamp DNA Mini Kit (QIAGEN, Düsseldorf, Germany) was used to extract genomic DNA from 400 μL peripheral blood. A NanoDrop 8000 (Thermo Scientific^TM^, Waltham, MA, United States) and agarose gel electrophoresis (AGE) were used to assess the quality and quantity of the DNA samples.

### Whole-Exome Sequencing and Screening for the Causative Gene

WES and screening were performed on a BGISEQ-500 machine (BGI, China) to identify the causative mutation (BGI, China) (Zhao et al., 2018a, b), as described in Supplementary Materials and Methods.

The candidate variant was verified by Sanger sequencing. The forward (5′-ATCTCCACTAAATCAATCACCCAT-3′) and reverse (5′-AGCCCTGTCATTACACCTCAAA-3′) primers were designed using Primer5 ver. 0.4.0.

### Species Conservation Analysis and Homology Protein Modeling

Evolutionary conservation of the candidate mutant amino acid site was assessed using the UniProt Browser^1^ and ClustalX2.1.

To explore the effects of the mutation on the molecular structure of IRF6, a homology model was created. The 467 aa full-length IRF6 was used as wild type IRF6 (WT IRF6). SWISS-MODEL (swissmodel.expasy.org) was used for molecular simulation (Biasini et al., 2004). We used IRF5 (3dsh.1. A) as the same template to create a model for WT residues 239–445 (identity 60.17%) and for mutant residues 221–394 (identity 52.98%). PyMOL software (pymol.org) was used for visualization.

### Plasmid Construction

The human full-length *IRF6* coding sequence (Ensembl transcript ID: ENST00000367021.8) was cloned into the pEGFP-C1 vector (Clontech, Mountain View, CA, United States) and designated GFP-IRF6. The human mutant *IRF6* coding sequence (p.Ile363ArgfsTer33) was generated from the human full-length *IRF6* plasmid by site-directed mutagenesis and designated GFP-IRF6 p.Ile363ArgfsTer395. And sequences above were also cloned into pCS2 vector for injection.

### Fish Rearing and Husbandry

All work with zebrafish (adult, larval, and embryonic) was performed according to protocols approved by the Institutional Animal Care and Use Committees at the University of Iowa. Breeding Danio rerio were maintained as described in the University of Iowa Animal Care Facility (Westerfield, 1993). Embryos were kept at 28.5°C and staged according to hours post fertilization (hpf) (Kimmel et al., 1995). The *irf6* mutant allele was zf2064, created by the Liao group (Li et al., 2017).

### Variant mRNA Synthesis and Embryo Microinjections

*Not*I-HF was used to linearize the plasmids (pCS2 backbone) containing target *IRF6* frameshift gene variant cDNAs. Linearized plasmids as a template, variant mRNA was synthesized using the SP6 mMESSAGE mMACHINE transcription kit (Ambion, Austin, TX, United States) by *in vitro* transcription. DNase I was used to digested the cDNA template, and the RNA Clean & Concentrator kit (Zymo Research, Irvine CA, United States) was used to purify the variant mRNA and a NanoDrop spectrophotometer to quantify it. Variant mRNAs were stored in −80°C. For embryo microinjections, ∼1 ng of the injection mix was delivered directly into the cytoplasm of one-cell staged embryos with variant mRNA diluted to a final concentration of 200 ng/μl. *LacZ* mRNA was injected as the negative control.

At 7 and 24 hpf, images were viewed using a Leica MZFL III (Solms, Germany) and acquired with a Q-imaging QIClick F-CLR-12 using Image pro (Media Cybernetics, Rockville, MD, United States).

Dominant negative IRF6, comprised of the DNA binding domain and the engrailed repressor domain (Sabel et al., 2009), was used to simulate the condition in another paradigm. At the one- to four-cell stages, embryos were injected with mRNA (approximately 4 nl at 60 ng/μl). Subsequently, some embryos were injected a second time with mRNA encoding a rescue construct, either the reference or patient variant of IRF6 (approximately 4 nl at 200 ng/μl). Each assay contained four groups of embryos, three of which were injected with *dnIRF6* mRNA alone, *dnIRF6* mRNA + rescue construct mRNA (double injection), or rescue construct alone; the fourth group was uninjected controls n = 50–350 embryos per group.

### Cell Culture

Human embryonic kidney epithelial cells (HEK-293FT) were maintained as monolayer cultures in Dulbecco’s modified Eagle’s medium (DMEM; Life Co., United States) and cultured in an incubator (37°C, 5% CO_2_).

### Transient Transfection

For transient transfections, cells were seeded into wells. After reaching >70% confluence at 12–24 h, HEK-293FT cells were transfected using Lipofectamine 3000^®^ Reagent (Invitrogen, Carlsbad, United States) following the manufacturer’s instructions. GFP positive cells were observed and counted under a fluorescence microscope after 36 or 48 h.

### Western Blotting

Thirty μg of cell lysates were separated by 10% SDS-PAGE under reducing conditions and transferred to PVDF membranes (Cwbiotech, China). Primary mouse anti-GFP antibody (Cell Signaling Technology, Danvers, MA, United States) at a dilution of 1:1000 was used to probe proteins. Membranes were then incubated with a fluorescent secondary antibody. The Odyssey^®^ LI-COR Imaging System (LI-COR Biotechnology, Lincoln, NE, United States) was used to visualize proteins.

### Protein Stability Assay

Transfected HEK-293FT cells were treated 36 h post-transfection with 20 μg/ml cycloheximide [CHX; (Sigma, St. Louis, MO, United States)] for up to 12 h, or with 10 μM MG132 (St. Louis, MO, United States) before CHX treatment for 30 min. GFP positive cells were calculated under a fluorescence microscope after treatment for 6 and 12 h and finally lysed and subjected to SDS-PAGE and western blotting.

## Results

### Phenotypic Descriptions of the Recruited Pedigree

**FIGURE 1:**
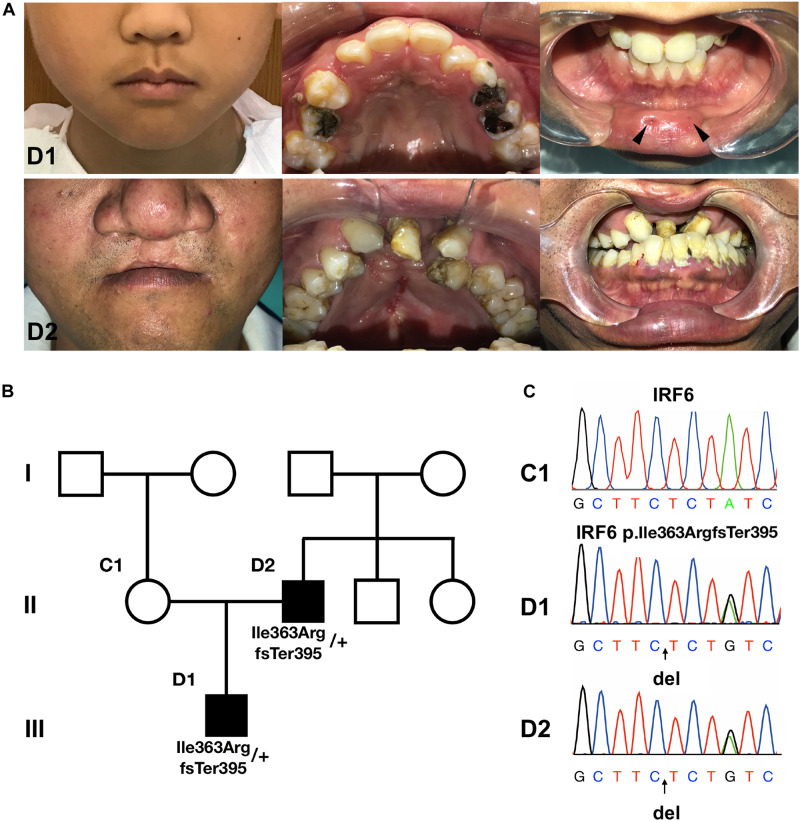
The pedigree information, phenotypes and Sanger sequencing. **(A,B)** The proband (D1) has bilateral lower lip pits (indicated by black arrows), shallow cleft palate and uvula fissa. His father (D2) has bilateral cleft lip and palate. His mother (C1) is healthy. **(C)** Sanger sequencing of the causative mutation. The mutations in D1 and D2 were heterozygous; C1 is the wild type. Arrows indicate the position of causative variants.

We recruited a three-generation Han Chinese family affected with VWS. The proband (D1) has bilateral lower lip pits, shallow cleft palate and uvula fissa. His father (D2) has a bilateral cleft lip and palate (Figures 1A,B). His mother (C1) is healthy. D1 and D2 underwent detailed oral and general physical examinations. D2 has a crossbite and both of his upper second incisors and the left first incisor are congenitally absent. D1 and C1 do not have severe malocclusion. We confirmed that the patients lacked other gross malformations, including cranio-skeletal development, external ear morphology and audition, eyes and vision, neuromuscular and motor systems, morphology of the long bones, cardiovascular system, and external genitals. Because of the cleft phenotype in both patients and lip pits in D1, we diagnosed this family with VWS pedigree.

C1 reported having no exposure during pregnancy to smoking, alcohol, radioactivity, or chemical teratogens, and not suffering any diseases. She did not report taking antibiotics or supplements with folic acid, iron agent, vitamin B_6_.

### WES and Variant Screening Identified a Novel IRF6 Frameshift Mutation

WES was performed on D1, D2, and C1 in this VWS family. Human GRCh37/hg19 was the reference genome. Detailed data and analysis were presented in Supplementary Materials and Methods, Supplementary Figure S1 and Supplementary Tables.

Using Sanger sequencing, we verified that the heterozygous mutation (c.1088-1091delTCTA) in *IRF6* was present in (D1 and D2) but not in C1 (Figure 1C). As this variant was not reported in the gnomAD database of human variants (queried on [2018.6.30]), we applied Sanger sequencing to 54 healthy samples (data not shown). No mutation in this position was found.

### Conservation Analysis and Modeling Indicate the Variant Is Deleterious

**FIGURE 2:**
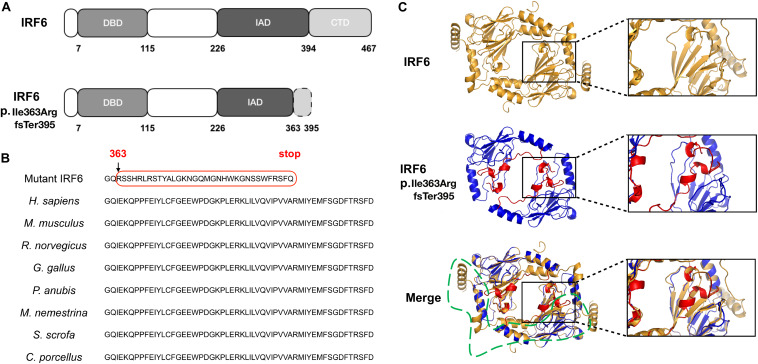
The position, conservation and homology models of the mutant protein. **(A)** The mutation was located in the IRF-association domain (IAD), p.Ile363ArgfsTer395, affecting a length of amino acids located from the end of the IAD to the entire C-terminal domain (CTD). **(B)** The residues from Ile363 to the end are evolutionarily conserved in *Homo sapiens*, *Mus musculus*, *Rattus norvegicus*, *Gallus gallus*, *Papio Anubis*, *Macaca nemestrina*, *Sus scrofa*, and *Cavia porcellus*. **(C)** Wild-type (residues 239–445), mutant (residues 221–394) and merged homology model. The frameshift in IRF6 p.Ile363ArgfsTer395 yields a stretch of 33 residues (i.e., RSSHRLRSTYALGKNGQMGNHWKGNSSWFRSFQ, labeled by the red box in Figure 2B) prior to a premature termination codon that are absent from the reference variant; the mutant IRF6 is missing much of the carboxy half of the protein, including 4 β-sheets and 3 α-helical regions (labeled by the green box in Figure 2C).

The frameshift of IRF6 at Ile363 affects a length of highly conserved amino acids located from the end of the IRF-association domain (IAD) to the entire C-terminal domain (CTD) (Figures 2A,B). Furthermore, a homology model for this region of IRF6 revealed significant difference in the structures between WT and mutant proteins (Figure 2C). Both the mimic modeling of WT or mutant IRF6 form homo-dimers. However, because of the existence of β-sheets at the carboxy terminus end of IRF6, IRF6 is predicted to have a more compact structure than IRF3. The frameshift in IRF6 p.Ile363ArgfsTer395 yields a stretch of 33 residues prior to a premature termination codon that are absent from the reference variant (Figures 2B,C). Finally, the mutant IRF6 is missing much of the carboxy half of the protein, including 4 β-sheets and 3 α-helical regions (Figure 2C), and including serine residues that must be phosphorylated for IRF6 to be activated (Oberbeck et al., 2019). There is thus a strong prediction that the protein lacks normal function.

### Functional Rescue of Zebrafish Periderm Rupture With Reference and Frameshift IRF6 Variants

**FIGURE 3:**
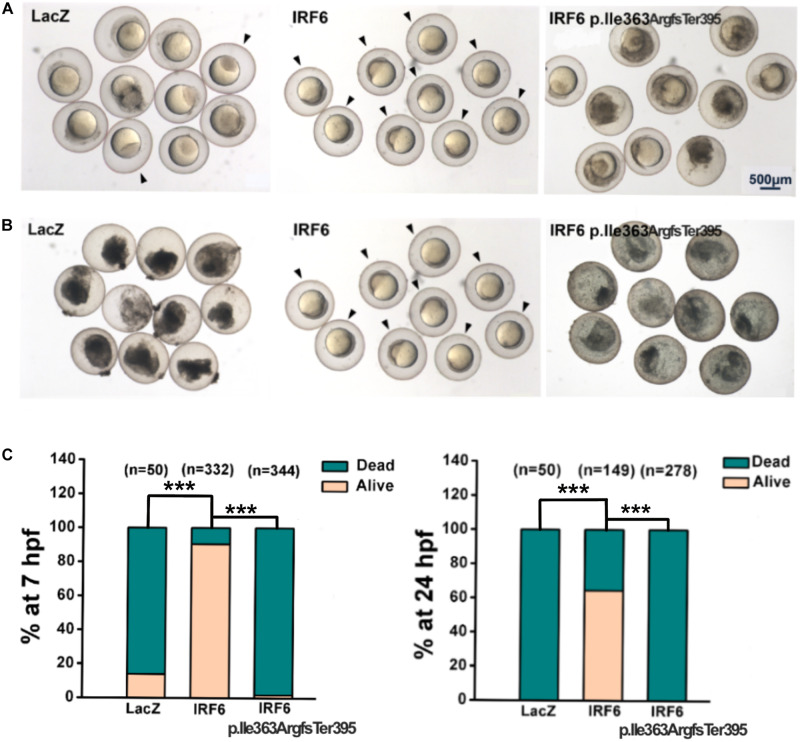
Rescue assay in maternal-null mutant *irf6* zebrafish embryos. The cDNAs for the LacZ, IRF6 reference variant (as positive control) and the p.Ile363ArgfsTer395 variant were engineered into a plasmid (separately), transcribed mRNA *in vitro*, and microinjected the mRNAs (separately) into maternal-null mutant *irf6* zebrafish embryos at the one-cell stage. **(A)** In 7 h, 14% of embryos injected with mRNA encoding LacZ were viable, 90.3% of embryos injected with mRNA encoding reference variant were viable and only 1.7% of embryos injected with the mRNA encoding frameshift variant were viable (indicated by black arrows); **(B)** In 24 h, 64.4% of embryos injected with mRNA encoding reference variant were viable (indicated by black arrows), none of embryos injected with the mRNA encoding LacZ, or frameshift variant were viable (***P < 0.001).

Maternal/zygotic-null mutant *irf6* zebrafish embryos have been used in a rescue paradigm to test the function of Irf6 variants detected in patients with OFC (Li et al., 2017). We deployed a similar assay except using maternal-null mutant *irf6* zebrafish embryos (i.e., the paternal genotype was wildtype). We found that uninjected maternal-null mutant *irf6* zebrafish embryos, and those injected with *LacZ* mRNA rupture around 7 h post fertilization (hpf), as reported for maternal/zygotic-null mutant *irf6* zebrafish embryos (Li et al., 2017). To compare the functions of the frameshift variant from the VWS family described here with the wildtype IRF6, we engineered the cDNAs for the *IRF6* reference variant and the p.Ile363ArgfsTer395 variant into a plasmid (separately), transcribed mRNA *in vitro*, and microinjected the mRNAs (separately) into maternal-null mutant *irf6* zebrafish embryos at the one-cell stage and assessed the periderm rupture phenotype as the embryos developed (Figure 3). As the positive control, we injected the human *IRF6* reference variant and found that 90.3% of injected embryos were viable at 7 hpf, and 64.4% appeared normal at 24 hpf. By contrast, only 1.7% of embryos injected with the frameshift variant were viable at 7 hpf. None were viable at 24 hpf. We observed comparable results in a rescue paradigm co-injecting mRNAs encoding a previously described dominant-negative variant of IRF6 and mRNAs encoding patient or reference variants of IRF6 (Sabel et al., 2009) (Supplementary Figure S2). These results suggest the frameshift protein could not play the same role as the WT IRF6 to help the periderm normally form.

### The Frameshift of IRF6 at Ile363ArgfsTer395 Causes Reduced Expression Levels

**FIGURE 4:**
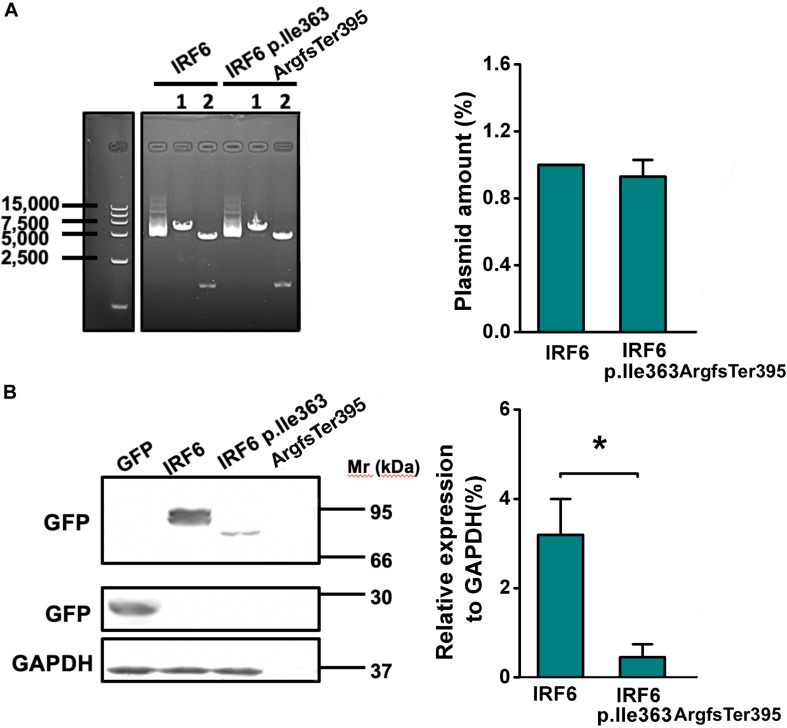
The frameshift of IRF6 at Ile363ArgfsTer395 causes reduced protein abundance level. **(A)** The plasmids expressing GFP-tagged versions of wildtype IRF6 and IRF6 p.Ile363ArgfsTer395 perform equal quantities. 1: single enzyme-cut linear plasmid; 2. Double enzyme-cut linear plasmid. **(B)** IRF6 p.Ile363ArgfsTer395 abundance levels were consistently seven- to eight-fold lower than those for WT IRF6. The difference was significant (*P < 0.05).

To further evaluate the function of the IRF6 Ile363 frameshift variant, HEK293FT cells were transfected with equal amount plasmids expressing GFP-tagged versions of wildtype IRF6 and IRF6 p.Ile363ArgfsTer395 (Figure 4). Western blot of cell lysates confirmed that IRF6 p.Ile363ArgfsTer395 abundance levels were consistently seven- to eight-fold lower than those for WT IRF6. Therefore, the protein abundance of IRF6 p.Ile363ArgfsTer395 is at greatly reduced levels in comparison to WT IRF6 *in vitro*.

### Frameshift of IRF6 at Ile363 Causes Its Proteasome-Dependent Degradation

**FIGURE 5:**
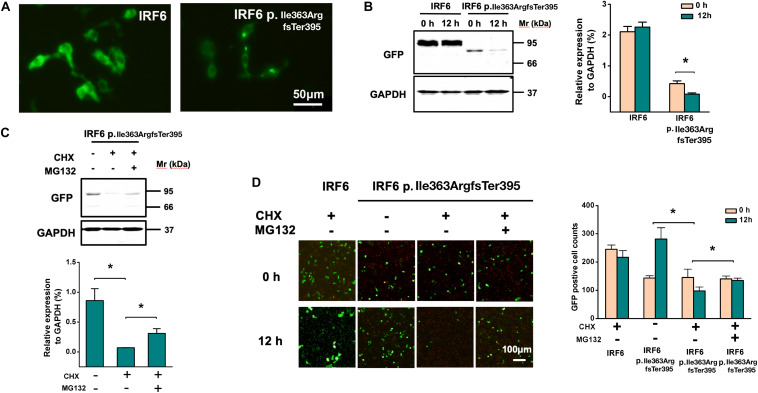
Frameshift of IRF6 at Ile363 causes its proteasome-dependent degradation. **(A)** Images of cells expressing IRF6 p.Ile363ArgfsTer395 under fluorescence presented much weaker GFP fluorescence, but several bright dots in cytoplasm, suggesting the existence of proteasome-dependent degradation. **(B)** Western blot of transfected HEK293FT cells lysis when treated at 36 h post-transfection with CHX. Over the 12-h time-course, no significant decline in WT IRF6 levels were observed but IRF6 p.Ile363ArgfsTer395 levels rapidly declined. The difference is significant (*P < 0.05). **(C)** Treated transfected HEK293FT cells with the proteasome inhibitor MG132 for 30 min prior to CHX addition. Western blot indicated that in cells treated with MG132, the loss of IRF6 p.Ile363ArgfsTer395 was attenuated modestly, the difference is significant (*P < 0.05). **(D)** GFP fluorescence observation of transfected HEK293FT cells when treated at 36 h post-transfection with CHX. Over the 12-h time-course, no significant decline in WT IRF6 levels were observed but IRF6 p.Ile363ArgfsTer395 levels rapidly declined. GFP fluorescence observed modestly attenuation when treated GFP-IRF6 p.Ile363ArgfsTer395 plasmid transfected HEK293FT cells with the proteasome inhibitor MG132 for 30 min prior to CHX addition (*P < 0.05).

Images of cells expressing IRF6 p.Ile363ArgfsTer395 under fluorescence presented much weaker GFP fluorescence. Both wild-type and p.Ile363ArgfsTer395 mutant IRF6 were expressed in the cytoplasm and in the nucleus. However, we saw several bright dots in the cytoplasm in p.Ile363ArgfsTer395 mutant-transfected cells (Figure 5A), suggesting proteasome-dependent degradation dots (Abe et al., 2013).

To establish whether the decreased abundance of IRF6 p.Ile363ArgfsTer395 was due to lower stability, transfected HEK293FT cells were treated at 36 h post-transfection with CHX to inhibit further *de novo* protein synthesis. Changes in WT IRF6 and IRF6 p.Ile363ArgfsTer395 levels were then measured over time by microscope observation and Western blot. Over the 12-h time-course, no significant decline in WT IRF6 levels were observed. In contrast, following CHX addition, IRF6 p.Ile363ArgfsTer395 levels rapidly declined (Figure 5B).

We also treated transfected HEK293FT cells with the proteasome inhibitor MG132 for 30 min prior to CHX addition. In cells treated with MG132, the loss of IRF6 p.Ile363ArgfsTer395 was attenuated modestly, as shown by Western blot (Figure 5C) and by GFP fluorescence (Figure 5D). This implies that the much higher levels of GFP signal in GFP-IRF6 transfected cells versus those in IRF6 p.Ile363ArgfsTer395 transfected cells is the result, at least in part, of proteasome degradation of the latter.

## Discussion

OFC and lower lip pits are the typical features of VWS but these phenotypes have variable expressivity: 12–15% of individuals with VWS present with isolated OFC (Leslie et al., 2016b), others present lip pits with or without OFC. Mutations in *IRF6* are the main cause of VWS and single nucleotide polymorphisms at its locus, 1q32, are associated with risk of non-syndromic OFC in previous studies (Park et al., 2007; Rahimov et al., 2008; Beaty et al., 2010; Sun et al., 2015).

We collected a VWS family in which the proband and his father display distinct phenotypes. However, at the first medical examination, the lower lip pits in the proband were ignored, thus the pedigree was misdiagnosed as non-syndromic cleft lip and/or palate. WES was applied to identify the candidate casual variants. When the frameshift variant of *IRF6* was screened out, there was a doubt that whether this pedigree was misdiagnosed. Therefore, they were recalled for the second-time physical examination, and finally diagnosed as VWS. And a frameshift of IRF6 at Ile363 was emerged as the most likely causal variant. Although the lip pits could not be examined in the father, the subclinical phenotypes cannot be totally excluded (Dixon et al., 2011). And because of the lip pits in the proband, his father should also be diagnosed as VWS. Nonetheless, we cannot exclude the possibility of oligogenic or polygenic models.

Using the IRF3 structure as a reference, we assessed the potential influence of the frameshift mutation on the structure of IRF6. The homo-dimer was speculated as an active conformation according to the structure of IRF3. A hydrophobic core would be formed when the IRF-association domain (IAD) and N- and C-terminal flanking autoinhibitory elements interact with each other (Qin et al., 2003). A compact structure with poor transactivation potential was believed to form when the DNA-binding domain interact with the C-terminal autoinhibitory element (Qin et al., 2003). Truncation of IRF3 at a position equivalent to Ile363 in IRF6 would lead to the loss of the C-terminal flanking autoinhibitory element.

It is possible that the IAD of IRF6 is inhibited by autoinhibitory elements in a similar way. Therefore, IRF6 p.Ile363ArgfsTer395 may adopt a less stable structure compared to WT, which is relatively open and extended conformation and hence potentially more likely to degradation. Of note, similar degradation has also been observed in other IRF6 truncations of carboxy-terminal domain, supporting this hypothesis (Kwa et al., 2015).

*Irf6* maternal-null zebrafish embryos provide a visual tool to assess the function of different variants and provide approaches to discussing the relationship among genotype, function influence and phenotype (Li et al., 2017). Therefore, we performed zebrafish rescue assay to evaluate the damaging effects of *IRF6* variants in our study. While 100% of uninjected mutants ruptured by 7 hpf, WT human *IRF6* mRNA rescued 90% of injected embryos at 7 hpf, with 64.4% of injected embryos surviving until 24 hpf. The frameshift *IRF6* mRNA rescued virtually none even at the earlier stage. Importantly, we found the same results using a rescue assay of embryos injected with mRNA encoding dominant-negative Irf6; thus, an *in vivo* test of IRF6 function can be carried out in any zebrafish (or frog) lab without the *irf6* mutant (Sabel et al., 2009). In summary, the IRF6 variant detected in a VWS patient could not exhibit the same function as WT IRF6 in zebrafish.

In addition to likely lacking normal function, the abundance of IRF6 p.Ile363ArgfsTer395 variant is at much lower level than the reference variant. In HEK293FT cells expressing GFP-tagged WT IRF6, the protein is detected in the cytoplasm and nuclear. By contrast, in those transfected with frameshift of IRF6 at Ile363, bright green dots are observed in the cytoplasm, indicating the aggregates of protein result from the proteasome being overwhelmed as it tries to degrade a misfolded protein (Abe et al., 2013). It has been previously reported that a frameshift variant of IRF6 truncated at its carboxy terminus encountered proteasome-dependent degradation (Kwa et al., 2015). To explore the mechanism effecting the level of frameshift IRF6 p.Ile363ArgfsTer395, CHX was applied to inhibit protein synthesis, and abundance levels of IRF6 p.Ile363ArgfsTer395 were monitored after a chase period. Such levels were lower than those of similarly transfected WT IRF6 in similarly treated cells, suggesting the IRF6 p.Ile363ArgfsTer395 is degraded faster than the WT protein. Inhibition of the proteasome led to partial recovery of IRF6 p.Ile363ArgfsTer395 levels, implicating the proteasome’s involvement in the degradation of IRF6 p.Ile363ArgfsTer395. It has been reported previously that IRF6 would be degraded by the proteasome in a cell cycle-dependent manner (Bailey et al., 2008). But it is unclear whether the degradation of IRF6 p.Ile363ArgfsTer395 occurs by the same mechanism as the cell cycle-dependent degradation of WT IRF6, although it is also by the proteasome. In contrast to IRF6, IRF1, another member of IRF family, is a short-lived protein. The proteasome-dependent degradation of IRF1 would be prevented by the removal of its C-terminal region. Therefore, the influence of the C-terminal region on the stability and degradation of IRF paralogs varies depending on the family member (Nakagawa and Yokosawa, 2000). Although the underlying mechanism is unclear, the greatly accelerated rate at which IRF6 p.Ile363ArgfsTer395 is degraded may partially explain why this family suffers VWS.

In our cellular assay, HEK cells were used to investigate the protein stability, which are pattern cells for general biology experiments, and sometimes involved in the investigations of OFC (Eshete et al., 2018). However, they are not necessarily the typically correct cell types for investigations of craniofacial phenotypes. Other cell types originated from craniofacial tissues such as GMSM-K should be used in further exploration.

In cells treated with MG132, levels of IRF6 p.Ile363ArgfsTer395 did not recover to the same levels as WT IRF6. It is possible that MG132 did not inhibit the proteasome completely. Alternatively, other degradation pathways may be involved. For instance, it is possible nonsense-mediated decay (NMD) of the mRNA encoding IRF6 p.Ile363ArgfsTer395 results in lower levels of protein being translated in comparison to in cells transfected with the mRNA encoding IRF6 reference variant. The existence of NMD in mammalian cells depends on the exon-exon junctions and generally degrades mRNAs that terminate translation more than 50–55 nucleotides upstream of a splicing-generated exon-exon junction (Jungwook and Lynne, 2011). In the *IRF6* frame-shift variant considered here, the protein is predicted to terminate in the beginning of the last exon, so this mutation may not elicit NMD. Further studies are necessary to assess the stability of the mutant RNA and explore other mechanisms that may influence protein stability.

Another reason the IRF6 p.Ile363ArgfsTer395 variant lacks function may be that it lacks the serine and threonine residues whose phosphorylation mediate dimerization, translocation into the nucleus, and regulation of transcription (Bailey et al., 2005, 2008). Activation of IRF6 appears to be regulated by phosphorylation at Ser-413 and Ser-424 by RIPK4 (Kwa et al., 2014; Oberbeck et al., 2019). The simulated structure not only indicates the loss of phosphorylation sites but also the change of 3D structure that could directly influence the function of IRF family members. Kwa et al. reported that truncation of IRF6 at Arg412 not only caused its rapid proteasome-dependent degradation, but also prevented the induction of its transactivator function by RIPK4 due to loss of phosphorylation sites (Kwa et al., 2015). In our research, p. Ile363ArgfsTer395 may suffer a similar mechanism.

In conclusion, we reported a new frameshift variant of IRF6 at Ile363 as a possible casual candidate in a VWS family. Species conservation analysis and homology protein modeling predicted the potential pathogenicity. *In vivo*, IRF6 p. Ile363ArgfsTer395 could not rescue *irf6* maternal-null mutant zebrafish embryos from eruption, resulting from the loss of normal periderm. *In vitro*, upon overexpression in cells, the IRF6 p. Ile363ArgfsTer395 was relatively unstable and was preferentially targeted to the proteasome in comparison to the WT IRF6.

Dominant negative effect is a common reason for the functional impairment of the mutant IRF6 (Sabel et al., 2009; de la Garza et al., 2013). But the less stable frameshift protein is not likely to have that effect. Therefore, haploinsufficiency is our favored mechanism in this VWS pedigree, in accordance with previous studies (Schutte et al., 1999; Kondo et al., 2002). Our study expands the casual variants spectrum for CLP and reports the potential mechanism. However, the genetic background for CLP is complex. For example, dominant *GRHL3* mutations were demonstrated to cause VWS and were associated with non-syndromic cleft-palate without cleft lip (Leslie et al., 2016b; Mangold et al., 2016; Eshete et al., 2018); however, there is also incompletely penetrant, as 46% individuals with mutations presented with apparently isolated CL/P and 10% were even asymptomatic. More exploration should be done in the future not only for the completion of casual variant spectrum, but also the possible mechanism for CLP and the relationship between genotypes and phenotypes. The polygenic background should be considered. In our pedigree, the two patients showed different phenotypes. We speculated that common variants might also contribute to the variability (Sun et al., 2015). In our sequencing results, except for *IRF6*, there are other gene variants that could possible contribute to the final phenotype, such as *ARHGAP29* (Letra et al., 2014) and *GLI3* (Gurrieri et al., 2007) (Supplementary Table S3). And more members from a pedigree should be analyzed for more information about the genotype and phenotype variety.

## Data Availability Statement

The datasets generated for this study can be found in the CNGBdb (https://db.cngb.org, Program No. CNP0001010).

## Ethics Statement

The participants provided their written informed consent to participate in this study or written informed consent was obtained from their guardian. This study was approved by the Human Research Ethics Committee of Peking University Hospital of Stomatology (PKUSSIRB-20150012). Informed consent was obtained from all the participants.

## Author Contributions

FC, JL, and RC made contributions to the conception or design of the work. MZ and JZ collected and contributed the main the data. MZ wrote the manuscript. HZ contributed to the manuscript revision. VI contributed to partial the data. WZ and WH contributed to sample collection and analysis. RC edited the manuscript.

## Conflict of Interest

The authors declare that the research was conducted in the absence of any commercial or financial relationships that could be construed as a potential conflict of interest.

## Footnotes

1 www.uniprot.org

## Supplementary Material

FIGURE S1 The variants with high frequency (MAF > 0.5%) were excluded (15,116 variants in D1 and 14,631 variants in D2 remained). Variants that were more likely to be pathogenic, such as non-synonymous variants, splice acceptor-site or donor-site mutations, inserts and deletions (Naming “Coding and Splicing” in Supplementary Figure S1D) were included (1,899 variants in D1 and 1,829 variants in D2 remained). The variants shared by D1 and D2 but not in C1 were selected, which reduced the number of candidate genes to 435. After excluding the variants with Phenolyzer, 17 variants remained. The literature on the individual variants and candidate genes was reviewed for potential significance related to the phenotype and possible deleterious effects on craniofacial structures.

FIGURE S2 Rescue paradigm in WT zebrafish embryos when co-injecting mRNAs encoding a dominant-negative variant of Irf6 (dnIRF6) and mRNAs encoding IRF6 p.Ile363ArgfsTer395 or reference variants of IRF6. At 8 h, 89.9% embryos were alive when they were uninjected; 31.3% embryos were alive when injected with mRNA encoding dnIRF6; 97.7 and 93.3% embryos were alive when injected, respectively, with mRNA encoding reference variants of IRF6 and IRF6 p.Ile363ArgfsTer395. 69% embryos were alive when co-injecting *dnIRF6* and reference *IRF6* mRNA. 12.2% embryos were alive when co-injecting mRNA encoding dnIRF6 and IRF6 p.Ile363ArgfsTer395. At 24 h, 86.2% embryos were alive when they were uninjected; 5.5% embryos were alive when injected with dominant-negative variant of *Irf6* mRNA; 94.4% and 80.0% embryos were alive when injected, respectively, with mRNA encoding reference variants of IRF6 and IRF6 p.Ile363ArgfsTer395. 21.6% embryos were alive when co-injecting mRNA encoding dnIRF6 and reference IRF6 mRNA. 0% embryos were alive when co-injecting mRNA encoding dnIRF6 and IRF6 p.Ile363ArgfsTer395.

TABLE S1 Summary of whole exome sequencing data and alignment.

TABLE S2 Summary statistics for identified variants.

TABLE S3 Candidates for the causative variant.

## References

[B1] Abe A. Takahashi-Niki K. Takekoshi Y. Shimizu T. Kitaura H. Maita H. (2013). Prefoldin plays a role as a clearance factor in preventing proteasome inhibitor-induced protein aggregation. *J. Biol. Chem.* 288 27764–27776. 10.1074/jbc.m113.476358 23946485 PMC3784693

[B2] Bailey C. M. Abbott D. E. Margaryan N. V. Khalkhali-Ellis Z. Hendrix M. J. C. (2008). Interferon regulatory factor 6 promotes cell cycle arrest and is regulated by the proteasome in a cell cycle-dependent manner. *Mol. Cell. Biol.* 28:2235. 10.1128/mcb.01866-07 18212048 PMC2268429

[B3] Bailey C. M. Khalkhali-Ellis Z. Kondo S. Margaryan N. V. Seftor R. E. B. Wheaton W. W. (2005). Mammary serine protease inhibitor (Maspin) binds directly to interferon regulatory factor 6: identification of a novel serpin partnership. *J. Biol. Chem.* 280 34210–34217. 10.1074/jbc.m503523200 16049006 PMC3175759

[B4] Beaty T. H. Murray J. C. Marazita M. L. Munger R. G. Ruczinski I. Hetmanski J. B. (2010). A genome-wide association study of cleft lip with and without cleft palate identifies risk variants near MAFB and ABCA4. *Nat. Genet.* 42 525–529. 20436469 10.1038/ng.580 PMC2941216

[B5] Biasini M. Bienert S. Waterhouse A. Arnold K. Studer G. Schmidt T. (2004). SWISS-MODEL: modelling protein tertiary and quaternary structure using evolutionary information. *Nucleic Acids Res.* 42 W252–W258. 10.1093/nar/gku340 PMC4086089 24782522

[B6] Bixler D. Poland C. Nance W. E. (1973). Phenotypic variation in the popliteal pterygium syndrome. *Clin. Genet.* 4 220–228. 10.1111/j.1399-0004.1973.tb01146.x 4203060

[B7] de la Garza G. Schleiffarth J. R. Dunnwald M. Mankad A. Weirather J. L. Bonde G. (2013). Interferon regulatory factor 6 promotes differentiation of the periderm by activating expression of Grainyhead-like 3. *J. Invest. Dermatol.* 133 68–77. 10.1038/jid.2012.269 22931925 PMC3541433

[B8] Dixon M. J. Marazita M. L. Beaty T. H. Murray J. C. (2011). Cleft lip and palate: understanding genetic and environmental influences. *Nat. Rev. Genet.* 12 167–178. 10.1038/nrg2933 21331089 PMC3086810

[B9] Eshete M. A. Liu H. Li M. Adeyemo W. L. Gowans L. J. J. Mossey P. A. (2018). Loss-of-function GRHL3 variants detected in african patients with isolated cleft palate. *J. Dent. Res.* 97 41–48. 28886269 10.1177/0022034517729819 PMC5755809

[B10] Ghassibe M. Revencu N. Bayet B. Gillerot Y. Vanwijck R. Verellen-Dumoulin C. (2004). Six families with van der Woude and/or popliteal pterygium syndrome: all with a mutation in the IRF6 gene. *J. Med. Genet.* 41:e15. 10.1136/jmg.2003.009274 PMC1735675 14757865

[B11] Gurrieri F. Franco B. Toriello H. Neri G. (2007). Oral–facial–digital syndromes: review and diagnostic guidelines. *Am. J. Med. Genet. Part A* 143A 3314–3323. 10.1002/ajmg.a.32032 17963220

[B12] Hammond N. L. Dixon J. Dixon M. J. (2019). Periderm: life-cycle and function during orofacial and epidermal development. *Semin. Cell Dev. Biol.* 91 75–83. 10.1016/j.semcdb.2017.08.021 28803895

[B13] Ingraham C. R. Kinoshita A. Kondo S. Yang B. Sajan S. Trout K. J. (2006). Abnormal skin, limb and craniofacial morphogenesis in mice deficient for interferon regulatory factor 6 (Irf6). *Nat. Genet.* 38 1335–1340. 10.1038/ng1903 17041601 PMC2082114

[B14] Jungwook H. Lynne E. M. (2011). Nonsense-mediated mRNA decay (n.d.) in animal embryogenesis: to die or not to die, that is the question. *Curr. Opin. Genet. Dev.* 21 422–430. 10.1016/j.gde.2011.03.008 21550797 PMC3150509

[B15] Kimmel C. B. Ballard W. W. Kimmel S. R. Ullmann B. Schilling T. F. (1995). Stages of embryonic development of the zebrafish. *Dev. Dyn.* 203 253–310. 10.1002/aja.1002030302 8589427

[B16] Koillinen H. Wong F. Rautio J. Ollikainen V. Karsten A. Larson O. (2001). Mapping of the second locus for the Van der Woude syndrome to chromosome 1p34. *Eur. J. Hum. Genet.* 9 747–752. 10.1038/sj.ejhg.5200713 11781685

[B17] Kondo S. Schutte B. C. Richardson R. J. Bjork B. C. Knight A. S. Watanabe Y. (2002). Mutations in IRF6 cause Van der Woude and popliteal pterygium syndromes. *Nat. Genet.* 32 285–289. 12219090 10.1038/ng985 PMC3169431

[B18] Kwa M. Q. Huynh J. Aw J. Zhang L. Nguyen T. Reynolds E. C. (2014). Receptor-interacting protein kinase 4 and interferon regulatory factor 6 function as a signaling axis to regulate keratinocyte differentiation. *J. Biol. Chem.* 289 31077–31087. 10.1074/jbc.m114.589382 25246526 PMC4223312

[B19] Kwa M. Q. Huynh J. Reynolds E. C. Hamilton J. A. Scholz G. M. (2015). Disease-associated mutations in IRF6 and RIPK4 dysregulate their signalling functions. *Cell Signal.* 27 1509–1516. 10.1016/j.cellsig.2015.03.005 25784454

[B20] Leslie E. J. Koboldt D. C. Kang C. J. Ma L. Hecht J. T. Wehby G. L. (2016a). IRF6 mutation screening in non-syndromic orofacial clefting: analysis of 1521 families. *Clin. Genet.* 90 28–34. 10.1111/cge.12675 26346622 PMC4783275

[B21] Leslie E. J. Liu H. Carlson J. C. Shaffer J. R. Feingold E. Wehby G. (2016b). A genome-wide association study of nonsyndromic cleft palate identifies an etiologic missense variant in GRHL3. *Am. J. Hum. Genet.* 98 744–754. 10.1016/j.ajhg.2016.02.014 27018472 PMC4833215

[B22] Letra A. Maili L. Mulliken J. B. Buchanan E. Blanton S. H. Hecht J. T. (2014). Further evidence suggesting a role for variation in ARHGAP29 variants in nonsyndromic cleft lip/palate. *Birth Defects Res. A Clin. Mol. Teratol.* 100 679–685. 10.1002/bdra.23286 25163644 PMC4623315

[B23] Li E. B. Truong D. Hallett S. A. Mukherjee K. Schutte B. C. Liao E. C. (2017). Rapid functional analysis of computationally complex rare human IRF6 gene variants using a novel zebrafish model. *PLoS Genet.* 13:e1007009. 10.1371/journal.pgen.1007009 28945736 PMC5628943

[B24] Little H. J. Rorick N. K. Su L. Baldock C. Malhotra S. Jowitt T. (2009). Missense mutations that cause Van der Woude syndrome and popliteal pterygium syndrome affect the DNA-binding and transcriptional activation functions of IRF6. *Hum. Mol. Genet.* 18 535–545. 10.1093/hmg/ddn381 19036739 PMC2638798

[B25] Malik S. Kakar N. Hasnain S. Ahmad J. Wilcox E. R. Naz S. (2010). Epidemiology of van der woude syndrome from mutational analyses in affected patients from Pakistan. *Clin. Genet.* 78 247–256. 10.1111/j.1399-0004.2010.01375.x 20184620

[B26] Malik S. Wilcox E. R. Naz S. (2014). Novel lip pit phenotypes and mutations of IRF6 in Van der Woude syndrome patients from Pakistan. *Clin. Genet.* 85 487–491. 10.1111/cge.12207 23713753

[B27] Mangold E. Bohmer A. C. Ishorst N. Hoebel A. K. Gultepe P. Schuenke H. (2016). Sequencing the GRHL3 coding region reveals rare truncating mutations and a common susceptibility variant for nonsyndromic cleft palate. *Am. J. Hum. Genet.* 98 755–762. 10.1016/j.ajhg.2016.02.013 27018475 PMC4833214

[B28] Nakagawa K. Yokosawa H. (2000). Degradation of transcription factor IRF-1 by the ubiquitin–proteasome pathway. *Eur. J. Biochem.* 267 1680–1686. 10.1046/j.1432-1327.2000.01163.x 10712599

[B29] Oberbeck N. Pham V. C. Webster J. D. Reja R. Huang C. S. Zhang Y. (2019). The RIPK4–IRF6 signalling axis safeguards epidermal differentiation and barrier function. *Nature* 574 249–253. 10.1038/s41586-019-1615-3 31578523

[B30] Park J. W. McIntosh I. Hetmanski J. B. Jabs E. W. Vander K. C. A. Wu-Chou Y. H. (2007). Association between IRF6 and nonsyndromic cleft lip with or without cleft palate in four populations. *Genet. Med.* 9 219–227. 17438386 10.1097/GIM.0b013e3180423cca PMC2846512

[B31] Peyrard-Janvid M. Leslie E. J. Kousa Y. A. Smith T. L. Dunnwald M. Magnusson M. (2014). Dominant mutations in GRHL3 cause van der woude syndrome and disrupt oral periderm development. *Am. J. Hum. Genet.* 94 23–32. 10.1016/j.ajhg.2013.11.009 24360809 PMC3882735

[B32] Qin B. Y. Liu C. Lam S. S. Srinath H. Delston R. Correia J. J. (2003). Crystal structure of IRF-3 reveals mechanism of autoinhibition and virus-induced phosphoactivation. *Nat. Struct. Mol. Biol.* 10 913–921. 10.1038/nsb1002 14555996

[B33] Rahimov F. Marazita M. L. Visel A. Cooper M. E. Hitchler M. J. Rubini M. (2008). Disruption of an AP-2 alpha binding site in an IRF6 enhancer is associated with cleft lip. *Nat. Genet.* 40 1341–1347. 10.1038/ng.242 18836445 PMC2691688

[B34] Richardson R. J. Dixon J. Malhotra S. Hardman M. J. Knowles L. Boot-Handford R. P. (2006). Irf6 is a key determinant of the keratinocyte proliferation-differentiation switch. *Nat. Genet.* 38 1329–1334. 10.1038/ng1894 17041603

[B35] Sabel J. L. d’Alencon C. O’Brien E. K. Van Otterloo E. Van Otterloo E. Lutz K. (2009). Maternal interferon regulatory factor 6 is required for the differentiation of primary superficial epithelia in Danio and Xenopus embryos. *Dev. Biol.* 325 249–262. 10.1016/j.ydbio.2008.10.031 19013452 PMC2706144

[B36] Schutte B. C. Basart A. M. Watanabe Y. Laffin J. J. S. Coppage K. Bjork B. C. (1999). Microdeletions at chromosome bands 1q32-q41 as a cause of Van der Woude syndrome. *Am. J. Med. Genet.* 84 145–150. 10.1002/(sici)1096-8628(19990521)84:2<145::aid-ajmg11>3.0.co;2-l 10323740

[B37] Sun Y. Huang Y. Yin A. Pan Y. Wang Y. Wang C. (2015). Genome-wide association study identifies a new susceptibility locus for cleft lip with or without a cleft palate. *Nat. Commun.* 6:6414. 10.1038/ncomms7414 25775280

[B38] Van Der Woude A. (1954). *Fistula labii* inferioris congenita and its association with cleft lip and palate. *Am. J. Hum. Genet.* 6 244–256. 13158329 PMC1716548

[B39] Westerfield M. (1993). *The Zebrafish Book.* Eugene, OR: Universuty of Oregon Press.

[B40] Zhao H. Zhang M. Zhong W. Zhang J. Huang W. Zhang Y. (2018a). A novel IRF6 mutation causing non-syndromic cleft lip with or without cleft palate in a pedigree. *Mutagenesis* 33 195–202. 10.1093/mutage/gey012 30053123

[B41] Zhao H. Zhong W. Leng C. Zhang J. Zhang M. Huang W. (2018b). A novel PTCH1 mutation underlies nonsyndromic cleft lip and/or palate in a Han Chinese family. *Oral Dis.* 24 1318–1325. 10.1111/odi.12915 29908092

[B42] Zucchero T. M. Cooper M. E. Maher B. S. Daack-Hirsch S. Nepomuceno B. Ribeiro L. (2004). Interferon regulatory factor 6 (IRF6) gene variants and the risk of isolated cleft lip or palate. *New Engl. J. Med.* 351 769–780. 15317890 10.1056/NEJMoa032909

