# Supplementary figures and images for: Functional Characterization of a Novel IRF6 Frameshift Mutation From a Van Der Woude Syndrome Family
Source: Front Genet. 2020 Jun 4;11:562. doi: 10.3389/fgene.2020.00562 (PMC7289175; doi:10.3389/fgene.2020.00562)

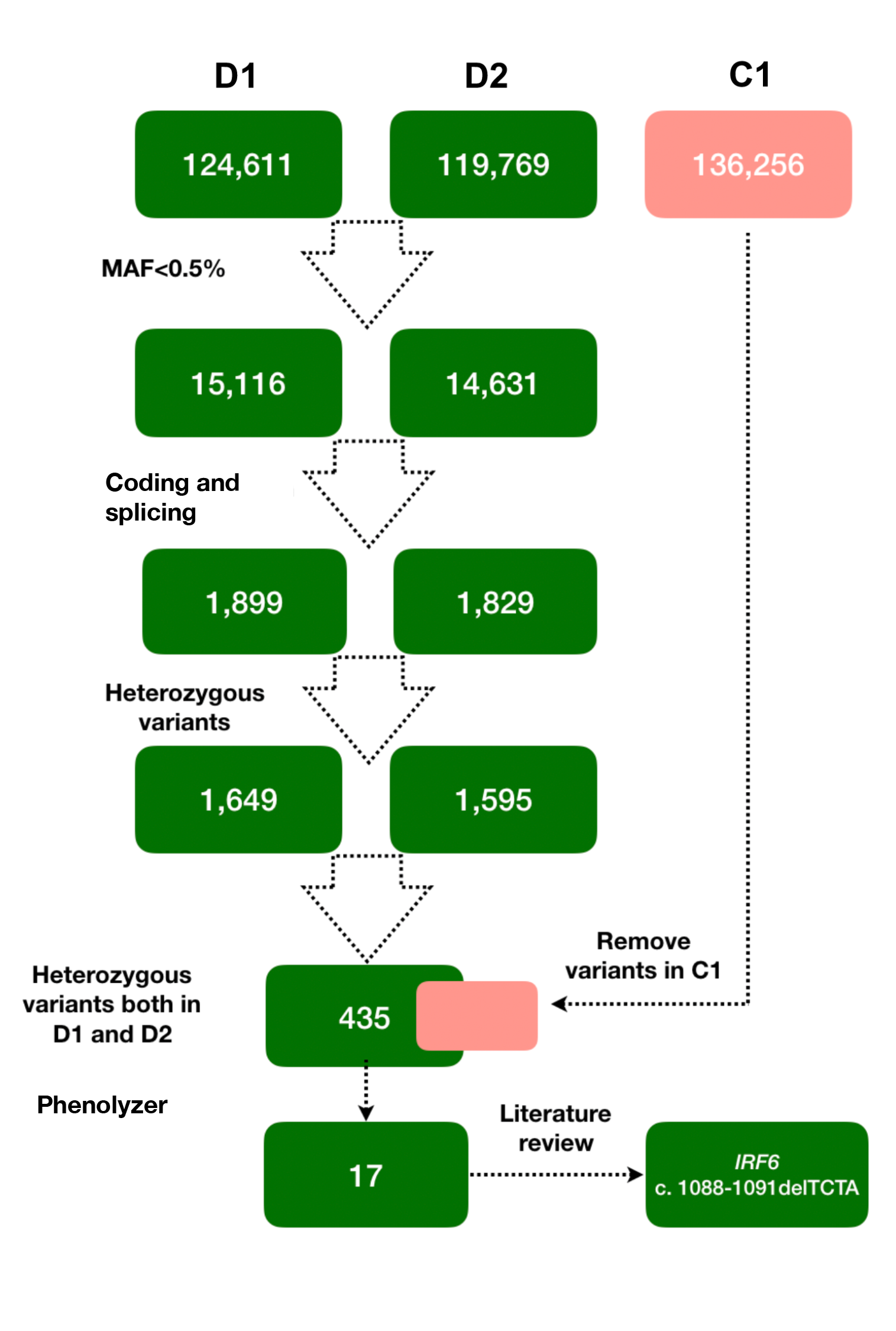

Supplement: FIGURE S1 — The variants with high frequency (MAF > 0.5%) were excluded (15,116 variants in D1 and 14,631 variants in D2 remained). Variants that were more likely to be pathogenic, such as non-synonymous variants, splice acceptor-site or donor-site mutations, inserts and deletions (Naming “Coding and Splicing” in Supplementary Figure S1D) were included (1,899 variants in D1 and 1,829 variants in D2 remained). The variants shared by D1 and D2 but not in C1 were selected, which reduced the number of candidate genes to 435. After excluding the variants with Phenolyzer, 17 variants remained. The literature on the individual variants and candidate genes was reviewed for potential significance related to the phenotype and possible deleterious effects on craniofacial structures. [file Image_1.TIF]

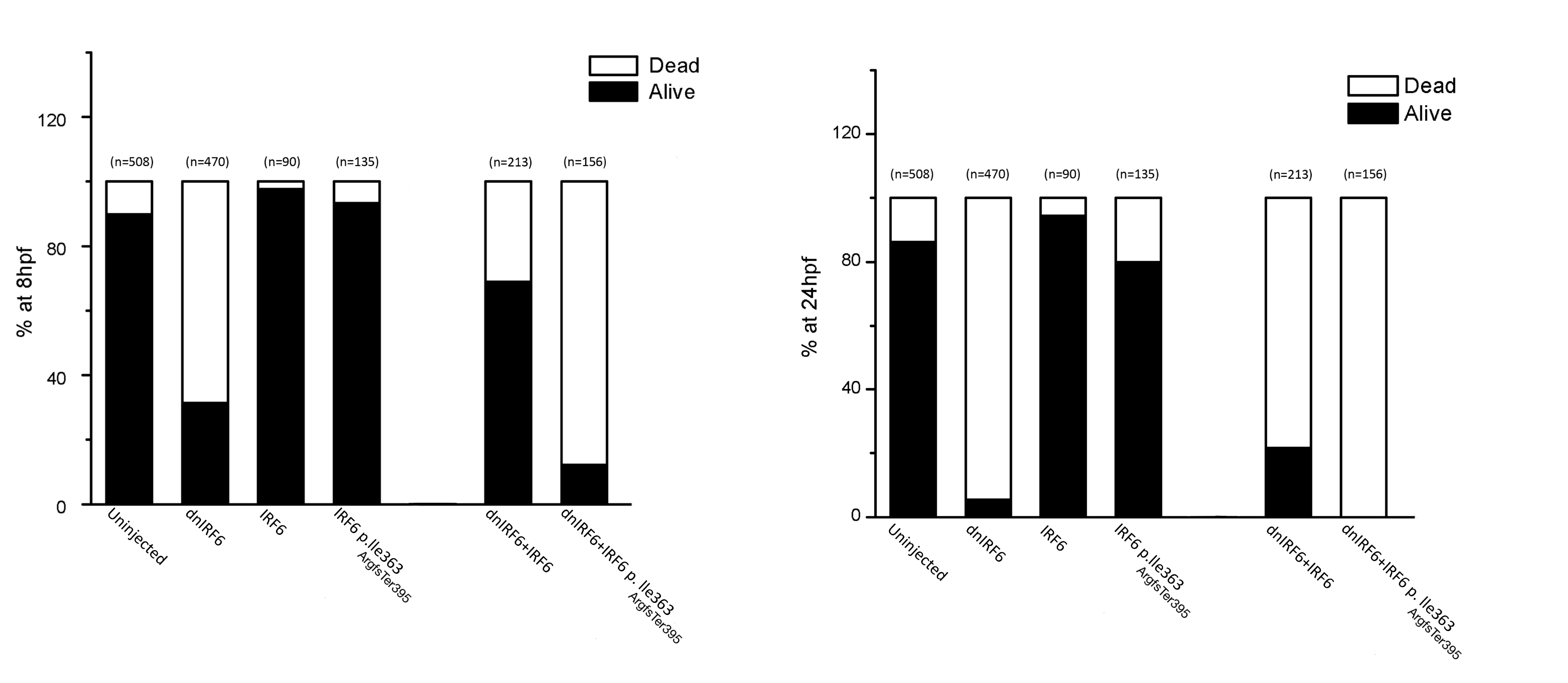

Supplement: FIGURE S2 — Rescue paradigm in WT zebrafish embryos when co-injecting mRNAs encoding a dominant-negative variant of Irf6 (dnIRF6) and mRNAs encoding IRF6 p.Ile363ArgfsTer395 or reference variants of IRF6. At 8 h, 89.9% embryos were alive when they were uninjected; 31.3% embryos were alive when injected with mRNA encoding dnIRF6; 97.7 and 93.3% embryos were alive when injected, respectively, with mRNA encoding reference variants of IRF6 and IRF6 p.Ile363ArgfsTer395. 69% embryos were alive when co-injecting dnIRF6 and reference IRF6 mRNA. 12.2% embryos were alive when co-injecting mRNA encoding dnIRF6 and IRF6 p.Ile363ArgfsTer395. At 24 h, 86.2% embryos were alive when they were uninjected; 5.5% embryos were alive when injected with dominant-negative variant of Irf6 mRNA; 94.4% and 80.0% embryos were alive when injected, respectively, with mRNA encoding reference variants of IRF6 and IRF6 p.Ile363ArgfsTer395. 21.6% embryos were alive when co-injecting mRNA encoding dnIRF6 and reference IRF6 mRNA. 0% embryos were alive when co-injecting mRNA encoding dnIRF6 and IRF6 p.Ile363ArgfsTer395. [file Image_2.TIFF]
