# Supplementary material for: Functional Characterization of a Novel IRF6 Frameshift Mutation From a Van Der Woude Syndrome Family
Source: Front Genet. 2020 Jun 4;11:562. doi: 10.3389/fgene.2020.00562 (PMC7289175; doi:10.3389/fgene.2020.00562)
Supplement: TABLE S1 — Summary of whole exome sequencing data and alignment. [file Table_1.docx]

**Supplemental Materials and Methods**

**Whole-exome sequencing and screening for the causative gene**

The clean data for each sample were mapped to the human reference genome (GRCh37/HG19) using the Burrows–Wheeler Aligner (BWA) (Oxford, England). SnpEff (<http://snpeff.sourceforge.net/SnpEff_manual.html>) was then used to perform annotation and prediction.

We included variants with a minor allele frequency ≤ 0.5% in public databases (the 1000 Genomes Project Asian, <http://www.1000genomes.org/>), excluded variants in the non-coding DNA sequence, including the 3'-UTR, 5'-UTR, downstream, upstream, intergenic, and intron regions and non-coding exon variants. Based on a single-gene dominant inheritance Mendelian model, each variant was evaluated for the potential to contribute to the VWS. Literature review was conducted for individual variants and candidate genes in order to figure out the potential significant relationships to the OFC and deleterious effects on craniofacial structures.

WES was performed on D1, D2 and C1 in this VWS family. Each sample yielded an average of 26.22 Gb of mappable, targeted exome sequences with a mean sequencing depth over 238-fold. 98.84% of the exonic regions were covered at least 4-fold (**Supplemental Table 1**).

After mapping to the human GRCh37/hg19 reference genome, 126,879 variants on average per person (including single nucleotide, multiple nucleotide, insertion, and deletion variants) were identified, among which 69,185.3 were heterozygous, and 57,693.3 were homozygous (**Supplemental Table 2**).

After annotation of variants, we applied a screening and filtration process to identify the candidate gene mutation (**Supplemental Figure 1**). Common variants (allele frequency of ≥ 0.5% in 1000 Genomes Project, Asian) were filtered out. Next, we identified variants locating in DNA coding sequencing region. Subsequently, because VWS showed a dominant inheritance model, we selected heterozygous variants that were shared by D1 and D2, and excluded the variants shared by C1 (435 variants). Next, we used the Phenolyzer screening tool (http://phenolyzer.usc.edu) (1) to examine previously reported genes with known roles in the pathogenesis of cleft lip and palate. Eventually, we noted c.1088-1091delTCTA in *IRF6* (NM_006147.3), predicting p.Ile363ArgfsTer395 and resulting in a frameshift mutation, as candidate casual variant.

# References

1. Yang H, Robinson PN, Wang K. Phenolyzer: phenotype-based prioritization of candidate genes for human diseases. *Nat Methods* (2015) 12(9):841-3.

**Supplemental Table 1** Summary of whole exome sequencing data and alignment

| Sample | Total effective yield (Gb) | Average sequencing depth (x) | Q20% | Q30% | Mapping rate on genome (%) | Mismatch rate in target region (%) | Fraction of target covered≥4x (%) | Fraction of target covered≥10x (%) |
| --- | --- | --- | --- | --- | --- | --- | --- | --- |
| D1 | 24.11 | 224.98 | 98.76 | 94.20 | 99.93 | 0.70 | 99.60 | 98.53 |
| D2 | 24.70 | 256.10 | 98.74 | 94.10 | 99.93 | 0.70 | 99.61 | 98.97 |
| C1 | 29.85 | 235.81 | 98.73 | 93.53 | 99.94 | 0.24 | 99.59 | 99.02 |
| Average | 26.22 | 238.96 | 98.74 | 93.94 | 99.93 | 0.55 | 99.60 | 98.84 |
