# Supplementary material for: Functional Characterization of a Novel IRF6 Frameshift Mutation From a Van Der Woude Syndrome Family
Source: Front Genet. 2020 Jun 4;11:562. doi: 10.3389/fgene.2020.00562 (PMC7289175; doi:10.3389/fgene.2020.00562)
Supplement: TABLE S2 — Summary statistics for identified variants. [file Table_2.docx]

**Supplemental Table 2** Summary statistics for identified variants

| Sample | Total variation | Heterozygotes | Homozygotes | Exonic | Intronic | Intergeneic | Splicing | Synonyomus | Stop-gain | Stop-loss | Missense |
| --- | --- | --- | --- | --- | --- | --- | --- | --- | --- | --- | --- |
| D1 | 124,611 | 66,891 | 57,720 | 39,562 | 85,049 | 3,128 | 2,305 | 11,143 | 84 | 40 | 10,498 |
| D2 | 119,769 | 64,564 | 55,205 | 38,950 | 80,819 | 3,125 | 2,285 | 11,152 | 88 | 40 | 10,386 |
| C1 | 136,256 | 76,101 | 60,155 | 41,570 | 94,686 | 3,516 | 2,368 | 11,259 | 89 | 38 | 10,303 |
| Average | 126,879 | 69,185 | 57,693 | 40,027 | 86,851 | 3,256 | 2,319 | 11,185 | 87 | 39 | 10,396 |
