# Supplementary material for: Functional Characterization of a Novel IRF6 Frameshift Mutation From a Van Der Woude Syndrome Family
Source: Front Genet. 2020 Jun 4;11:562. doi: 10.3389/fgene.2020.00562 (PMC7289175; doi:10.3389/fgene.2020.00562)
Supplement: TABLE S3 — Candidates for the causative variant. [file Table_3.docx]

| **Gene name** | **Function** | **Transcript ID and mutation** | **Frequency in population from 1000 Genomes Project** | **dbSNP** | **Impact** | **SIFT** | **Reference base** | **Observation base** |
| --- | --- | --- | --- | --- | --- | --- | --- | --- |
| **IRF6** | **frameshift_variant** | **NM_006147.3:p.Ile363ArgfsTer33/c.1088_1091delTAGA** | - | - | **HIGH** | - | **CTCTA** | **C** |
| TNS1 | missense_variant | NM_022648.4:p.Pro474Ser/c.1420C>T | - | - | MODERATE | 0.44(T) | G | A |
| TBX4 | missense_variant | NM_018488.2:p.Ala59Thr/c.175G>A | 0.001 | rs80164240 | MODERATE | - | G | A |
| SEMA3A | missense_variant | NM_006080.2:p.Lys689Glu/c.2065A>G | - | - | MODERATE | 1(T) | T | C |
| RMND5A | missense_variant | NM_022780.3:p.Glu212Lys/c.634G>A | - | - | MODERATE | 0.24(T) | G | A |
| NEK2 | missense_variant | NM_002497.3:p.Gln153Pro/c.458A>C | - | - | MODERATE | 0.04(D) | T | G |
| MYOG | missense_variant | NM_002479.5:p.Arg147His/c.440G>A | 0.003 | rs141840619 | MODERATE | 0.42(T) | C | T |
| MEIS3 | missense_variant | NM_020160.2:p.Asp155Asn/c.463G>A | 0.004 | rs145236300 | MODERATE | 0.09(T) | C | T |
| LOXHD1 | splice_region_variant | NM_144612.6:c.3914C>T | - | - | LOW | - | G | A |
| GNAI3 | splice_region_variant | NM_006496.3:c.162T>C | 0.003 | rs182530939 | LOW | - | T | C |
| GLI3 | missense_variant | NM_000168.5:p.Ala161Thr/c.481G>A | - | - | MODERATE | 0.17(T) | C | T |
| FOLH1 | missense_variant | NM_004476.1:p.His475Tyr/c.1423C>T | 0.001 | rs61886492 | MODERATE | 1(T) | G | A |
| FOLH1 | missense_variant | NM_004476.1:p.Pro160Ala/c.478C>G | 0 | rs138510346 | MODERATE | 0.02(D) | G | C |
| DOLK | missense_variant | NM_014908.3:p.Ile484Val/c.1450A>G | 0 | rs147630977 | MODERATE | 0.09(T) | T | C |
| DHCR7 | splice_region_variant | NM_001163817.1:c.99G>A | 0.003 | rs140748737 | LOW | 0.13(T) | C | T |
| CYP26B1 | splice_region_variant | NM_019885.3:c.862G>A | - | - | LOW | - | C | T |
| ARHGAP29 | missense_variant | NM_004815.3:p.Val418Ile/c.1252G>A | 0.004 | rs148959325 | MODERATE | 1(T) | C | T |

**Supplemental Table 3** Candidates for the causative variant
